# Nutritional Status and Dietary Assessment in Kidney Transplant Recipients

**DOI:** 10.3390/nu18071145

**Published:** 2026-04-02

**Authors:** Martyna Magalska, Sylwia Małgorzewicz

**Affiliations:** Department of Clinical Nutrition, Medical University of Gdańsk, 80-210 Gdańsk, Poland; m.magalska@gumed.edu.pl

**Keywords:** kidney transplantation, protein intake, nutritional status, drug-induced diabetes mellitus

## Abstract

**Background:** Proper nutrition plays a crucial role in post-kidney transplantation care, influencing graft function, body composition, and the risk of metabolic complications. Protein intake is of particular importance due to its role in preserving muscle mass and preventing protein energy wasting. **Objective:** This study aimed to assess dietary intake, with emphasis on protein consumption, and to analyze its associations with nutritional status, anthropometric indices, and metabolic complications in kidney transplant recipients. **Materials and Methods:** A cross-sectional study was conducted in 71 adult kidney transplant recipients. Dietary intake was assessed using a 24 h dietary recall and the FFQ-6 questionnaire. Anthropometric measurements were performed, and multiple indices of body composition and central obesity were calculated. Associations between dietary intake, anthropometric parameters, age, and kidney graft function were analyzed. **Results:** Mean BMI was within the upper normal range; however, a high prevalence of central adiposity was observed. Age was positively correlated with indices of visceral obesity (ABSI, AVI, WHtR, and CI). Protein intake was positively associated with calf circumference, indicating a relationship with muscle mass preservation. Dietary analysis revealed excessive sodium intake and insufficient intake of potassium, calcium, vitamin D, and unsaturated fatty acids. Post-transplant diabetes mellitus was present in 25.35% of participants. **Conclusions:** Kidney transplant recipients frequently present with unfavorable body composition and dietary imbalances that are not adequately reflected by BMI alone. Comprehensive nutritional assessment and individualized dietary counseling are important strategies that may help reduce the risk of metabolic complications and support long-term transplant outcomes.

## 1. Introduction

Organ transplantation is an established therapeutic strategy involving the transfer of an organ, tissue, or cells between individuals or within the same individual to restore impaired physiological functions. The most common procedure in clinical practice is allogeneic transplantation (allotransplantation), which allows organ transfer between genetically non-identical individuals. Kidney transplantation is frequently preceded by renal replacement therapy, including hemodialysis and peritoneal dialysis. An alternative approach is kidney transplantation performed in the predialysis period, known as pre-emptive transplantation (PET) [1]. Nutritional requirements vary across treatment stages and play an important role in clinical outcomes. In end-stage chronic kidney disease, the risk of cardiovascular disease, including ischemic heart disease and peripheral vascular disease, increases progressively and is further elevated in patients receiving dialysis, irrespective of the dialysis modality. In kidney transplant recipients, cardiovascular risk is significantly lower than during dialysis; however, it remains elevated and requires regular monitoring and appropriate dietary management. Increasingly, transplant recipients present with obesity (body mass index > 30 kg/m^2^). During the first year following successful kidney transplantation, patients often experience an approximate 10% increase in body weight. Contributing factors include improved appetite, relaxation of dietary restrictions, and immunosuppressive therapy. Metabolic complications such as hyperglycemia, dyslipidemia, and hypertension are common and are further exacerbated by obesity.

Post-transplant diabetes mellitus (PTDM) is a frequent metabolic complication in kidney transplant recipients and in recipients of other solid organ transplants. The diagnosis of PTDM requires the exclusion of pre-existing type 1 and type 2 diabetes mellitus or metabolic syndrome prior to transplantation. In patients treated with an immunosuppressive regimen consisting of tacrolimus, mycophenolic acid, and corticosteroids, PTDM occurs in approximately 20–30% of cases. Calcineurin inhibitors, including tacrolimus, represent the cornerstone of immunosuppressive therapy and contribute to impaired insulin secretion from pancreatic β-cells [2,3]. Vincenti et al. reported a higher diabetogenic potential of tacrolimus compared with cyclosporine in transplant recipients [4]. PTDM is associated with an increased risk of cardiovascular disease and peripheral vascular disease and is linked to more than a 60% higher risk of graft failure and nearly a 90% higher risk of mortality.

In this context, systematic assessment of nutritional status and detailed evaluation of dietary intake in kidney transplant recipients constitute essential components of comprehensive post-transplant care. Appropriately tailored nutritional interventions may reduce the risk of metabolic complications, support graft function, and improve long-term clinical outcomes. In addition to conventional anthropometric measures, novel indices such as A Body Shape Index (ABSI), Abdominal Volume Index (AVI), Body Roundness Index (BRI), Conicity Index (CI), and Waist-to-Height Ratio (WHtR) are increasingly recognized as valuable tools for assessing body fat distribution and visceral adiposity, providing more precise information on cardiometabolic risk than BMI alone, particularly in populations with altered body composition such as kidney transplant recipients.

Therefore, this study aimed to assess dietary intake, with particular emphasis on protein consumption, and to evaluate its associations with nutritional status, including novel anthropometric indices of body fat distribution, and metabolic complications in kidney transplant recipients.

## 2. Materials and Methods

### 2.1. Study Design and Participants

The study was conducted among 71 kidney transplant recipients from the Department of Nephrology, Transplantology, and Internal Medicine in Gdańsk. Ethical approval for the study was obtained from the Bioethical Committee of Gdańsk Medical University (no NKBBN/343/2018, 10 October 2018). The study was carried out between 30 November 2024 and 31 July 2025.

Inclusion criteria comprised confirmed kidney transplantation status, age ≥ 18 years, and written informed consent to participate in the study. Patients were excluded in cases of refusal to participate or incomplete medical records.

All participants underwent a standardized nutritional and medical interview that included questions regarding:

- time elapsed since transplantation,
- cause and number of kidney transplants,
- presence of steroid-induced diabetes,
- type of immunosuppressive therapy,
- serum creatinine concentration and eGFR,
- type of dialysis preceding transplantation,
- presence of lipid disorders (hypercholesterolemia).

### 2.2. Characteristics of the Study Group

The study group consisted of 71 kidney transplant recipients from deceased donors. Pre-emptive transplantation was performed in 8.45% of cases. The group included 32 women (45.1%) and 39 men (54.9%). Participants’ age ranged from 19 to 77 years (mean: 49 ± 14.3 years). The mean time since transplantation was 28.8 ± 52.17 months (range: 0.23–228 months).

In the study population, the cause of kidney failure was identified based on medical history. The causes of chronic kidney disease in the study population included: glomerulonephritis (18.57%), autosomal dominant polycystic kidney disease (ADPKD; 10.00%), autoimmune diseases (2.82%), congenital nephrotic syndrome (5.70%), Alport syndrome (2.86%), diabetic nephropathy (8.57%), genetic diseases and congenital anomalies (4.29%), gout (1.43%), and rare causes such as Dense Deposit Disease (DDD), autosomal dominant tubulointerstitial kidney disease-UMOD type (ADTKD-UMOD), and multiple myeloma (MM; 1.43%).

Immunosuppressive therapy consisted of either a triple-drug regimen (*n* = 52) or a dual-drug regimen (*n* = 19). Calcineurin inhibitors were administered to most patients: tacrolimus was used in 89.85% of cases, cyclosporine in 10.15%, and everolimus in 5.35%. As a second antiproliferative or adjunctive agent, mycophenolate mofetil was most commonly used (92.86%), followed by azathioprine (1.79%). All patients received glucocorticoids.

### 2.3. Anthropometric Measurements

Anthropometric measurements included body weight, height, and circumferences of the waist, hips, upper arm, and calf. Based on these measurements, the following indices were calculated:

BMI (Body Mass Index);

ABSI (A Body Shape Index), incorporating waist circumference, BMI, and height to predict mortality risk;

AVI (Abdominal Volume Index), estimating abdominal volume based on height and waist and hip circumferences;

BRI (Body Roundness Index), reflecting body fat amount and distribution;

CI (Conicity Index), based on waist circumference, body weight, and height, used to assess visceral fat distribution;

BAI (Body Adiposity Index), estimating total body fat based on hip circumference and height;

RPI (Reciprocal Ponderal Index), describing the relationship between height and the cube root of body mass;

WHt^0.5R (Waist-to-Height^0.5 Ratio), expressing the ratio of waist circumference to the square root of height.

All indices were calculated using appropriate mathematical formulas based on Gomes-Neto et al. [5].

### 2.4. Analysis of Nutrient Intake

In a group of 71 patients, nutrient intake was assessed using a 24 h dietary recall. The dietary recall was conducted by a trained dietitian using a standardized interview protocol. Portion sizes were estimated using household measures and food portion photographs. Diet quality and characteristic dietary patterns were evaluated using the FFQ-6 (Food Frequency Questionnaire-6). Dietary data were analyzed with Nuvero software.

### 2.5. FFQ-6 Questionnaire

The FFQ-6 (Food Frequency Questionnaire-6) was used to assess diet quality and identify characteristic dietary patterns, and estimate the prevalence of nutrient deficiencies in kidney transplant recipients. This validated, Polish-language qualitative tool consists of two main sections, ensuring simplicity and standardization of administration.

The first section includes demographic information: respondent sex, date of birth, and date of questionnaire completion, allowing contextualization of results in statistical analyses.

The main section comprises 62 food item groups organized into eight thematic categories reflecting the typical structure of the Polish diet. The food list was developed based on nutritional databases and modeled on the calibrated FIVeQ questionnaire, taking into account seasonality, regional preferences, and commonly consumed foods. The categories included: sweets and snacks; dairy products; bread and cereal products; fats and spreads; meat and meat products; fish and eggs; vegetables and legumes; fruits and beverages.

For each of the 62 items, respondents selected one of six frequency categories referring to habitual intake over the previous 12 months: (1) never or almost never, (2) once a month or less, (3) several times a month, (4) several times a week, (5) once daily, or (6) several times daily. Respondents were instructed to consider all meals and snacks consumed both at home and outside the home, without estimating portion sizes, focusing exclusively on frequency.

### 2.6. 24-h Dietary Recall

The 24 h dietary recall is a retrospective qualitative–quantitative method of dietary assessment based on a detailed description of all foods, dishes, and beverages consumed during the preceding 24 h. This method enables evaluation of individual or group dietary intake, focusing on quantity, quality, meal timing, and preparation methods, allowing calculation of nutrient intake and assessment of individual dietary components.

### 2.7. Laboratory Measurements

In all patients, serum creatinine concentration was measured using routine laboratory method as part of standard clinical care. Estimated glomerular filtration rate (eGFR) was calculated using the CKD-EPI (Chronic Kidney Disease Epidemiology Collaboration) equation.

### 2.8. Statistical Analysis

Statistical analyses were performed using JASP software (Version 0.95) and Microsoft Excel. Descriptive statistics are presented as mean ± standard deviation (SD) for continuous variables. The normality of data distribution was assessed using the Shapiro–Wilk test. Comparisons between groups were conducted using Student’s *t*-test for independent or paired samples when parametric assumptions were met. In cases of non-normal data distribution, the Mann–Whitney U test or the Wilcoxon signed-rank test was applied. The Kruskal–Wallis test was used for comparisons involving more than two groups. Levene’s test was used to assess the assumption of homogeneity of variances; a *p*-value < 0.05 was considered indicative of a violation of this assumption.

Correlations between variables were assessed using Pearson’s correlation coefficient (*r*), which evaluates the strength and direction of linear relationships. A *p*-value < 0.05 was considered statistically significant.

Basic measures of central tendency and variability were calculated for the study population. Statistical data are presented as mean, standard deviation, median, and range for all 71 participants, including both sexes and a wide age range.

## 3. Results

### 3.1. Anthropometric Assessment

The mean body mass index (BMI) was 24.9 ± 4.34 kg/m^2^, with a median value of 24.7 kg/m^2^, indicating that the study group was at the upper limit of the normal weight range (reference range: 18.5–24.9 kg/m^2^). The wide BMI range (16.9–37.1 kg/m^2^) reflects substantial heterogeneity within the study population, encompassing individuals from underweight to class II obesity. The results are presented in Table 1.

#### 3.1.1. Indices of Central Obesity

WHt^0.5R (Waist-to-Height^0.5 Ratio)

The mean WHt^0.5R was 7.1 ± 0.97 (median: 7.0), demonstrating an approximately normal distribution, with nearly identical mean and median values. The range of 4.9–9.8 was relatively narrow, indicating homogeneity of measurements within the study population. At present, no universally established cut-off values defining increased metabolic or oncological risk are available for this index.

WHR (Waist-to-Hip Ratio)

The mean WHR was 0.96 ± 0.10 (median: 0.90; range: 0.7–1.4). WHR reflects body fat distribution, with lower values indicating a more favorable health profile. Values below 0.85 in women and below 0.90 in men are considered indicative of the absence of abdominal obesity. The observed mean value of 0.96 suggests elevated central fat accumulation in part of the study population, while the wide range reflects substantial heterogeneity among participants.

WHtR (Waist-to-Height Ratio)

The mean WHtR was 0.544 ± 0.073 (median: 0.54; range: 0.36–0.78). WHtR is a sensitive indicator of metabolic risk, with values below 0.50 considered normal and values ≥ 0.50 associated with increased risk of type 2 diabetes and cardiovascular disease. The mean WHtR observed in this cohort exceeds the recommended threshold.

#### 3.1.2. Body Shape and Volume Indices

ABSI (A Body Shape Index)

The mean ABSI was 0.083 ± 0.007 (median: 0.083), showing a symmetric distribution with a narrow range (0.062–0.100). The low standard deviation indicates limited interindividual variability. No standardized clinical cut-off values are currently defined for ABSI.

AVI (Abdominal Volume Index)

The mean AVI was 17.4 ± 4.77 (median: 16.6), with a slightly right-skewed distribution. The wide range (7.9–30.8) reflects considerable variability in abdominal volume. Higher values indicate greater abdominal adiposity; however, no universally accepted cut-off values are available.

BRI (Body Roundness Index)

The mean BRI was 4.25 ± 1.60 (median: 4.08; range: 1.20–10.00), demonstrating a moderately right-skewed distribution. Values between 3.4 and 5.5 are associated with the lowest mortality risk and a favorable body shape, values below 3.4 indicate excessive leanness, and values above 6.9 are indicative of abdominal obesity with increased mortality risk. The mean BRI falls within the range classified as no statistically significant risk (3.41–4.44) or the lower range of moderate risk (4.45–6.91). The wide range and high maximum value suggest the presence of a subgroup with elevated risk.

CI (Conicity Index)

The mean CI was 1.30 ± 0.12 (median: 1.29; range: 0.96–1.54), showing an approximately normal distribution. A CI value of 1.0 corresponds to a cylindrical body shape, whereas 1.73 represents a fully biconical shape. Values above 1.25 are commonly used as a threshold for abdominal obesity. The observed mean slightly exceeds this cut-off.

#### 3.1.3. Obesity and Body Composition Indices

BAI (Body Adiposity Index)

The mean BAI was 25.0 ± 6.32 (median: 24.5), with a very wide range (9.2–47.2), indicating pronounced heterogeneity in estimated body fat percentage. Reference ranges are approximately 21–33% for women and 8–25% for men.

RPI (Reciprocal Ponderal Index)

The mean RPI was 41.2 ± 2.53 (median: 41.2; range: 35.1–46.5), indicating a symmetric distribution. Reference values typically range from 35 to 45, with lower values reflecting higher adiposity. The low standard deviation suggests homogeneity of this parameter and the absence of extreme outliers.

#### 3.1.4. Circumference Measurements

Calf Circumference

The mean calf circumference was 35.53 ± 3.40 cm (median: 35 cm; range: 28–43 cm), an important indicator of lower limb muscle mass and sarcopenia risk. Reference values for healthy adults are 33–42 cm for women and 35–45 cm for men.

Waist Circumference

The mean waist circumference was 92.46 ± 12.88 cm (median: 91 cm; range: 63–124 cm). Cut-off values for abdominal obesity are ≥80 cm for women and ≥94 cm for men (increased risk), and ≥88 cm for women and ≥102 cm for men (high risk).

Mid-Upper Arm Circumference (MUAC)

The mean MUAC was 28.19 ± 3.85 cm (median: 28 cm; range: 16–37 cm). Reference values for adults are approximately 31.3 cm for men and 28.5 cm for women. Values below 23 cm are commonly used to indicate undernutrition.

Hip Circumference

The mean hip circumference was 96.46 ± 11.07 cm (median: 98 cm; range: 63–132 cm). Reference values for women typically range from 90 to 100 cm.

#### 3.1.5. Laboratory Parameters

The mean estimated glomerular filtration rate (eGFR, CKD-EPI) was 45.9 mL/min/1.73 m^2^ (median: 45; range: 9–89). The mean serum creatinine concentration in the study population of 71 kidney transplant recipients was 1.9 ± 0.95 mg/dL (median: 1.7 mg/dL; range: 0.7–5.5 mg/dL).

Sex-based analysis revealed significant differences in body composition and fat distribution among kidney transplant recipients (Table 2 and Table 3). Men had higher body weight (+15.1%), waist circumference (+4.9%), and calf circumference (+3.4%), reflecting greater overall body mass and a predominantly android pattern of fat distribution. In contrast, women exhibited slightly higher BMI (+3.3%) and hip circumference (+2.3%), consistent with a gynoid pattern of fat distribution.

Waist circumference exceeded reference values more frequently in women. Among body shape indices, men showed higher ABSI, AVI, and CI values, indicating greater central fat accumulation, whereas women had higher BRI and BAI values, reflecting higher overall adiposity. WHtR and WHt^0.5R values were comparable between sexes.

### 3.2. Body Mass Index and Kidney Graft Function

In the study population, the estimated glomerular filtration rate (eGFR, CKD–EPI) was compared between two groups of patients stratified by BMI. The mean eGFR values were 46.2 and 46.8 mL/min/1.73 m^2^ in patients with normal BMI and in those with overweight or obesity, respectively. In both groups, mean eGFR values fell within the range corresponding to moderately decreased kidney function (KDIGO stage G3a, 45–59 mL/min/1.73 m^2^).

The standard deviation of eGFR was higher in the normal BMI group (±21.01) compared with the overweight/obesity group (±18.1) (*p* = 0.983), but no significant correlation between BMI and kidney graft function was observed.

The distribution of body mass index (BMI) in the study group of 71 participants was analyzed and classified into seven nutritional status categories according to the standard WHO classification. The largest proportion of participants had normal body weight (47.89%). The second largest category was overweight, comprising 35.21% of the study population.

**Figure 1:**
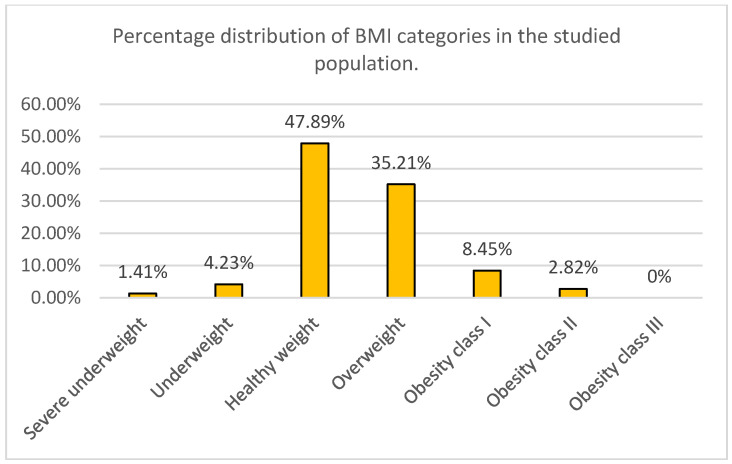
BMI categories in the study population.

Overall, undernutrition (emaciation and underweight) was observed in 5.64% of participants, while excessive body weight (overweight and all degrees of obesity) was present in 46.48%. Obesity of varying degrees was identified in 11.27% of the study group, with no cases of class III (morbid) obesity. The results are presented in Figure 1.

The mean BMI was also analyzed according to diabetes status and compared with patients without diabetes. The analysis included patients with type 1 diabetes, type 2 diabetes, and drug-induced diabetes. The highest mean BMI was observed in the group with type 2 diabetes, followed by the drug-induced diabetes group, while the lowest mean BMI was recorded in patients with type 1 diabetes.

One-way ANOVA showed no statistically significant differences in BMI values according to diabetes type (F(3,67) = 1.40; *p* = 0.252; ω^2^ = 0.016). The results are presented in Table 4.

### 3.3. Analysis of Body Weight Changes

**Figure 2:**
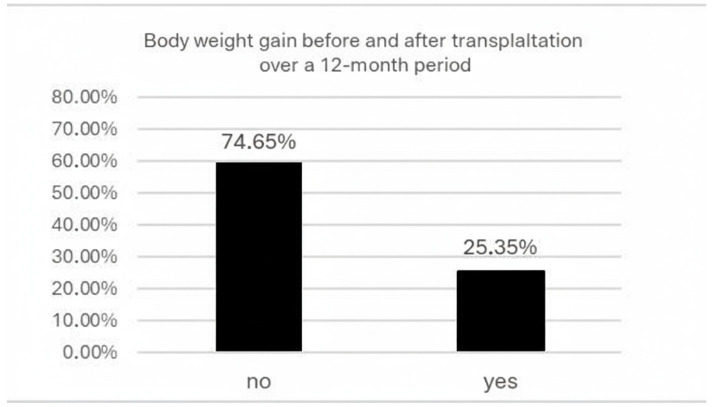
Body weight gain during the first 12 months after kidney transplantation.

Changes in body weight before and after kidney transplantation over the last 12 months are presented in Figure 2. A total of 74.65% of respondents presented no significant increase in body weight during this period, while 25.35% presented weight gain after transplantation. In addition, 63.38% of participants had no decrease in body weight, whereas 36.62% demonstrated a reduction in body weight after transplantation. Weight changes (both loss and gain) ranged from 0 to 45 kg.

More than 12 months after transplantation, both decreases and increases in body weight were observed, with a wide range of changes, from 2 to 30 kg for weight loss and from 3 to 40 kg for weight gain. A total of 9.86% of patients moved from normal body weight to overweight or obesity, while 11.27% transitioned from overweight or obesity to normal body weight.

In patients assessed 2–3 months after transplantation, body weight loss ranged from 2 to 45 kg over the 12 months prior to transplantation and the first 3 months post-transplantation. Weight gain in this subgroup ranged from 2 to 8 kg. Among patients in the early post-transplant period, 42.42% had a BMI outside the normal range.

Overall, 74.65% of patients did not report weight gain and 63.38% did not report weight loss during the analyzed period (Figure 2). Weight gain was reported by 25.35% of participants, while weight loss was reported by 36.62%, with changes ranging from 2 to 45 kg. Transitions between BMI categories occurred in 9.86% of patients moving from normal weight to overweight or obesity and in 11.27% moving from overweight or obesity to normal weight.

### 3.4. FFQ-6 Results

The highest consumption frequency was observed for vegetables and legumes (3.42 ± 0.68), followed by dairy products and eggs (3.05 ± 0.78) and meat and fish (2.92 ± 0.79) The data are presented in Table 5.

Relatively low mean values were recorded for energy drinks, alcohol, and other alcoholic beverages (1.20 ± 0.54), indicating very infrequent consumption. Beverages overall had a mean value of 2.40 ± 1.10, suggesting variability in consumption patterns within this category. Sweets and snacks, fats, and cereal products showed similar moderate levels of intake, with mean values of 2.30 ± 0.72, 2.50 ± 0.70, and 2.71 ± 0.60, respectively.

In summary, the diet of the study group was characterized by frequent inclusion of vegetables, balanced consumption of dairy and protein-rich products (meat, fish, eggs), and limited intake of alcoholic and energy drinks. This pattern indicates a tendency toward health-conscious dietary behaviors; however, the relatively low intake of unsaturated fats, cereal products, and fruits may suggest the need for further dietary optimization in relation to nutritional recommendations.

### 3.5. Analysis of Nutrient Intake in the Study Population

As shown in Table 6, energy and macronutrient intakes showed wide interindividual variability, with generally lower intake of polyunsaturated fatty acids and dietary fiber, and low vitamin D intake.

### 3.6. Assessment of Diet According to BMI

In the study population, the intake of energy, protein, fat, and selected minerals was analyzed by dividing participants into two categories based on body mass index (BMI).

In the group with a BMI of 16.9–24.9, corresponding to underweight and normal body weight, the mean daily energy intake was 1551.2 ± 538.1 kcal. The standard deviation indicates substantial variability in energy intake within this group. Mean protein intake was 75.17 ± 22.6 g/day, and mean fat intake was 56.4 ± 27.4 g/day.

Among participants with overweight and obesity (BMI 25.0–37.1), a slightly higher mean daily energy intake was observed (1607.6 kcal), accompanied by an even larger standard deviation (±573.9 kcal). Mean protein intake in this group was slightly lower at 73.59 ± 25.9 g/day, while fat intake was comparable to the normal BMI group, averaging 56.09 ± 24.1 g/day.

In the overweight and obese group (BMI 25.0–37.1), higher mean intakes of carbohydrates (212.4 ± 96.3 g/day) and potassium (2597.7 ± 1843.2 mg/day) were observed compared with the underweight and normal-weight group. Sodium intake was similar in both groups (2004.7 ± 723.0 mg/day). In contrast, mean calcium intake (515.6 ± 288.0 mg/day) and phosphorus intake (986.8 ± 391.1 mg/day) were slightly lower in participants with higher BMI. The results described above did not differ significantly between groups stratified by BMI.

### 3.7. Assessment of Diet According to Type of Diabetes

Mean intake and standard deviation of selected nutrients were compared across four groups: patients without diabetes, with type 1 diabetes, type 2 diabetes, and drug-induced diabetes (Table 7).

Patients with drug-induced diabetes had the highest mean energy intake (1758.9 kcal), as well as the highest intake of protein (78.8 g), fat (61.2 g), and carbohydrates (225.6 g). In contrast, patients with type 2 diabetes showed the lowest mean intake of energy (1341.7 kcal), fat (45.1 g), and carbohydrates (171.3 g), while patients with type 1 diabetes had the lowest mean protein intake (60.8 g). Patients without diabetes had intermediate energy intake (1543.4 kcal).

Mean calcium intake was low in all groups, with the lowest values observed in patients with type 1 diabetes (337.5 mg). Sodium intake was highest in the type 1 diabetes group (2315.5 mg) and lowest in the type 2 diabetes group (1830.9 mg). Potassium and phosphorus intake varied across groups, with the highest mean potassium and phosphorus intake observed in patients without diabetes.

Across all groups, large standard deviations were observed for most nutrients, indicating substantial heterogeneity in dietary intake, particularly in patients without diabetes and those with drug-induced diabetes.

### 3.8. Correlations of the Analyzed Parameters with Age and Protein Intake

Age was significantly correlated with ABSI (R = 0.389; *p* < 0.001), AVI (R = 0.324; *p* = 0.006), WHtR (R = 0.359; *p* = 0.002), and CI (R = 0.424; *p* < 0.001).

A statistically significant positive correlation was observed between protein intake and calf circumference (r = 0.260; *p* = 0.029). Higher protein intake was associated with greater calf circumference, a recognized marker of skeletal muscle mass which may reflect a relationship with skeletal muscle mass.

A strong positive correlation was found between WHtR and BAI (r = 0.659; *p* < 0.001) and between CI and WHR (r = 0.638; *p* < 0.001), confirming that both measures characterize central fat distribution.

## 4. Discussion

The nutritional status of kidney transplant recipients, including diet quality, represents a key component of long-term post-transplant care and is associated with graft function as well as the risk of metabolic and cardiovascular complications. In the present study, we assessed the relationships between selected anthropometric parameters, indices of body fat distribution, and dietary intake, with particular emphasis on protein consumption.

### 4.1. Anthropometric Characteristics

In the present study, no significant correlation was observed between the duration of kidney graft function and body mass index (BMI). This finding suggests that time elapsed since transplantation alone is not directly associated with body weight changes in kidney transplant recipients. Similar observations have been reported by other authors, who indicate that post-transplant weight changes are more strongly associated by lifestyle factors, immunosuppressive therapy, and dietary habits than by graft vintage itself [6]. The lack of a significant association may also be related to the heterogeneity of the study population with respect to time since transplantation.

Likewise, no significant correlation was found between age and BMI. This contrasts with trends observed in the general population, where BMI often increases with advancing age. In kidney transplant recipients, this finding may be explained by the influence of the underlying disease, long-term pharmacotherapy, and individualized dietary recommendations [5]. Importantly, the absence of an age and BMI association further suggests that BMI may not be a sufficiently sensitive indicator for assessing age-related changes in body composition in this population.

### 4.2. Protein Intake and Anthropometric Indicators of Muscle Mass

Adequate protein intake is considered an important factor associated with muscle mass preservation in kidney transplant recipients, particularly during post-transplant recovery and in the prevention of protein energy wasting (PEW) [7,8]. In the present study, a statistically significant positive association was observed between protein intake and calf circumference, suggesting the potential usefulness this simple anthropometric measure as an indicator of nutritional adequacy and skeletal muscle mass. However, it should be noted that the observed correlation between protein intake and calf circumference was relatively weak, and although statistically significant, it may be influenced by potential confounding factors such as age, sex, physical activity, and kidney function; therefore, its clinical relevance should be interpreted with caution.

Calf circumference has been widely validated as a surrogate marker of skeletal muscle mass and total body protein reserves, especially in patients with chronic kidney disease and after kidney transplantation [7]. In a cohort study by Kosoku et al. [7], protein intake was positively associated with changes in skeletal muscle mass index (SMI) during the first year after transplantation, while insufficient protein intake (<0.72 g/kg ideal body weight/day) was associated with lower muscle mass recovery. These findings are consistent with the present results and may indicate that patients with larger calf circumference, reflecting greater muscle mass, tend to consume higher amounts of dietary protein [9].

### 4.3. BMI, Time Since Transplantation, and Protein Intake

It should be acknowledged that body mass index (BMI), despite its widespread use, has important limitations in kidney transplant recipients, as elevated values may reflect increases in muscle mass, adiposity, or total body water. In this population, fluid retention and alterations in body composition may contribute to misclassification when BMI is used as the sole indicator of nutritional status. Therefore, BMI should be interpreted with caution and complemented by more precise assessment methods that enable differentiation between fat mass, lean tissue, and hydration status [8,9]. In the present study, BMI was not significantly associated with time since transplantation. This finding contrasts with data from long-term observational studies suggesting that BMI may be associated with graft survival and function over extended follow-up periods.

Bellini et al. [6], in a 5-year observational study including 396 kidney transplant recipients, demonstrated that BMI significantly affected graft survival and function at 2 and 4 years post-transplantation, while not influencing patient survival. The absence of a significant association in the present study may be attributable to the relatively short observation period, the predominance of patients with normal body weight, and the inclusion of a substantial proportion of individuals in the early post-transplant period.

Similarly, no significant relationship was observed between BMI and protein intake. This result differs from the findings of Yang et al. [8], who reported higher BMI values in patients consuming ≥1.4 g protein/kg body weight/day at three months post-transplantation. However, those authors emphasized that the increase in BMI reflected recovery of body mass after transplantation rather than excessive fat accumulation. Collectively, these findings indicate that BMI alone may be insufficient for evaluating qualitative changes in body composition after kidney transplantation.

### 4.4. Age and Central Fat Distribution Indices: ABSI and AVI

Age-related redistribution of adipose tissue toward the visceral compartment is a well-documented phenomenon, largely independent of total body mass [6,10]. In the present study, age was positively associated with ABSI, indicating progressive central obesity with advancing age among kidney transplant recipients.

Large population-based studies confirm the prognostic value of ABSI. Christakoudi et al. [10], analyzing data from the European EPIC cohort, demonstrated that ABSI is an independent predictor of all-cause mortality, regardless of BMI, smoking status, or physical activity. Similarly, Zhang et al. [11] reported a significant association between higher ABSI values and increased risk of hyperlipidemia, with each unit increase in ABSI corresponding to a 24% higher risk in fully adjusted models.

A significant positive correlation between age and AVI was also observed, reflecting increasing abdominal fat volume with age. Ramírez-Manent et al. [12] identified AVI as a strong predictor of metabolic syndrome and insulin resistance in large working populations, while Yan et al. [13] demonstrated that age significantly modifies the relationship between visceral fat indices and the risk of chronic kidney disease.

### 4.5. Age, WHtR, and Conicity Index (CI)

WHtR is widely recommended as a simple screening marker of cardiometabolic risk and has been shown to outperform BMI in the assessment of central obesity [14]. In the present study, age was positively correlated with WHt^0.5R, indicating increasing central fat accumulation with advancing age in kidney transplant recipients.

An even stronger association was observed between age and the conicity index (CI), suggesting that CI may be particularly sensitive to age-related changes in body shape and fat distribution. Dai et al. [15], analyzing data from the NHANES cohort, demonstrated a strong association between CI and both kidney stone risk and all-cause mortality, with each 0.1 unit increase in CI corresponding to a 51% increase in mortality risk. These findings suggest the potential clinical utility of CI in kidney transplant populations.

### 4.6. Interrelationships Among Anthropometric Indices

Strong correlations between WHtR and BAI, as well as between CI and WHR, indicate that these indices describe related aspects of fat distribution. WHtR primarily reflects central obesity, whereas BAI estimates total body adiposity; therefore, their strong association is expected and consistent with previous reports [15,16].

The very strong correlation between hip circumference and BAI has a methodological basis, as hip circumference is a key component of the BAI formula. Gao et al. [16], analyzing large prospective cohorts from China and the UK Biobank, demonstrated that higher BAI values were independently associated with an increased risk of chronic kidney disease, underscoring the clinical relevance of this index.

### 4.7. Sex-Based Differences in Anthropometric Indices

Sex-related differences in fat distribution are well documented in the literature [17]. In the present study, women exhibited higher BAI and BRI values, whereas men showed higher ABSI, AVI, and CI values, reflecting a predominance of peripheral fat accumulation in women and central obesity in men.

These findings are consistent with the study by Jao et al. [17], who reported significantly higher BAI values in women and stronger associations between central obesity indices and metabolic risk in men. Such differences should be considered in both research and clinical practice when assessing nutritional status and metabolic risk in kidney transplant recipients.

### 4.8. Diabetes After Kidney Transplantation

Diabetes mellitus (DM) is common after kidney transplantation (KTx), and it may also develop de novo as post-transplant diabetes mellitus (PTDM). Immunosuppressive therapy—particularly glucocorticoids (GCS), calcineurin inhibitors (CNI), and mTOR inhibitors (sirolimus, SIR; everolimus, EVR)—is an established risk factor for PTDM [18]. In this study, dietary intake differed by diabetes type, which is relevant for nutritional management. It should be emphasized, however, that the results of subgroup analyses according to diabetes type should be interpreted with caution, as some subgroups, particularly patients with type 1 diabetes, included a small number of participants, thereby limiting statistical reliability and precluding firm conclusions.

Patients with drug-induced diabetes (including PTDM/NODAT) had the highest energy intake, whereas the lowest intake was observed in patients with type 2 diabetes. However, energy intake in all groups remained below the recommended 30–35 kcal/kg/day in the stable post-transplant period. Inadequate energy intake in kidney transplant recipients (KTR) has been associated with protein energy wasting (PEW) and potentially poorer graft outcomes [19].

Protein intake was highest in patients with drug-induced diabetes and lowest in those with type 1 diabetes, remaining below recommended levels for the stable post-transplant phase. Current guidance suggests slightly higher protein intake in patients with post-transplant diabetes (0.8–0.9 g/kg/day) compared with those without diabetes (0.6–0.8 g/kg/day), potentially due to glycemic benefits. Clemente et al. reported that nutritional intervention improved glycemic control and lipid parameters in kidney transplant recipients with diabetes, particularly in those receiving continuous subcutaneous insulin infusion (CSII) [18,20].

Carbohydrate intake was highest in patients with drug-induced diabetes and lowest in those with type 2 diabetes. Given the role of carbohydrates in postprandial hyperglycemia, low-carbohydrate dietary approaches have been proposed to improve glycemic control and support weight management in KTR. Current international consensus recommends low-carbohydrate dietary patterns, lifestyle modification, and increased physical activity in transplant candidates with dysglycemia [19,21].

Fat intake was comparable across groups. However, evidence suggests that Mediterranean-style dietary patterns, particularly those rich in *n*-3 polyunsaturated fatty acids (PUFA), may reduce the risk of PTDM and improve metabolic outcomes. The dietary *n*-6:*n*-3 ratio has also been identified as an important qualitative parameter [19,22].

Sodium intake exceeded KDIGO recommendations (<2000 mg/day) in most groups, while potassium intake remained below WHO recommendations (3900 mg/day). These imbalances are clinically relevant, as potassium homeostasis is frequently disrupted in KTR and should be individualized according to graft function and clinical status [19,23]. Phosphorus intake is closely related to dietary protein and CKD stage, and should be interpreted accordingly [24].

Previous studies have reported limited differences in macronutrient distribution between patients with and without post-transplant diabetes, highlighting the need for further prospective studies and more specific dietary recommendations for this population, as current KDIGO guidelines do not provide detailed nutritional guidance for kidney transplant recipients [19,22,25,26].

BMI was lowest in patients with type 1 diabetes and highest in those with type 2 diabetes, and BMI trajectories have been identified as important predictors of progression to type 2 diabetes [27,28].

In PTDM/drug-induced diabetes, mean BMI was 25.4 ± 4.99 kg/m^2^; evidence indicates that pre-transplant BMI and post-transplant weight gain are independent PTDM risk factors, consistent with mixed mechanisms of insulin resistance and impaired insulin secretion [29,30]. Contemporary literature also reports the “double diabetes” phenomenon and an evolving type 1 diabetes phenotype, although the present type 1 subgroup remained within the normal BMI range [27].

### 4.9. Diet

Mean protein intake in the overall cohort was 74.4 ± 23.95 g/day, corresponding to 1.04 g/kg/day, with substantial interindividual variability, potentially reflecting both restrictive and high-intake dietary patterns relevant to graft workload. Higher protein intake (>1.2 g/kg/day) has been associated with a faster decline in eGFR in some cohorts, whereas increased protein intake in the early post-transplant period has also been linked to improved graft function; therefore, optimal long-term protein targets may depend on renal function assessed at one year after transplantation [8,26,31]. In addition, dietary patterns with a higher proportion of plant-based protein sources have been associated with nephroprotective effects in populations with chronic kidney disease [32].

Total fat intake was corresponding to a lower proportion of energy than commonly recommended (25–35%). Total fat intake was lower than commonly recommended (25–35% of total energy). Previous studies indicate that fat intake may increase in the early post-transplant period and remain elevated in the long term in some cohorts, while many patients follow general population guidelines due to the lack of transplant-specific recommendations [15,33].

Saturated fatty acid (SFA) intake slightly exceeded recommended levels. SFA restriction is emphasized in KDIGO and diabetes-related guidelines, and evidence suggests potential associations between specific SFA (e.g., palmitic acid) and graft and cardiovascular risk in kidney transplant recipients (KTR), while branched-chain fatty acids (BCFA) may differ compared with healthy controls [19,34,35].

Intake of monounsaturated (MUFA) and polyunsaturated fatty acids (PUFA) was below recommended levels. Despite this, increased serum MUFA concentrations observed in the early post-transplant period may reflect enhanced endogenous synthesis under insulin-resistant conditions. Current dietary recommendations therefore emphasize increasing the proportion of MUFA and PUFA within total fat intake [19,24,35]. Additionally, reduced omega-3 intake and an unfavorable *n*-6:*n*-3 ratio, typical of Western dietary patterns, have been reported in KTR and are associated with higher risk of graft failure and all-cause mortality [19,24,35,36].

Mineral intake showed considerable variability. Potassium and calcium intake were frequently below recommended levels, while sodium intake substantially exceeded KDIGO and KDOQI recommendations. These imbalances are consistent with previous studies and may contribute to adverse cardiovascular and graft-related outcomes [13,37,38,39]. Phosphorus intake may also be of concern, particularly due to the presence of phosphate additives in processed foods.

Vitamin intake patterns indicated adequate intake of vitamins A and C but insufficient intake of vitamins D and K [16]. B-vitamin intake was generally adequate for niacin and vitamin B6 but lower for folate and vitamin B12, which may contribute to hyperhomocysteinemia and increased cardiovascular risk, particularly in patients treated with mycophenolate mofetil [40].

Mean cholesterol intake remained within recommended ranges, although dietary lipid quality remains important due to immunosuppression-associated dyslipidemia. Dietary fiber intake was at the lower limit of recommendations and may be clinically relevant, as higher fiber intake has been associated with reduced cardiovascular mortality in chronic kidney disease and improved metabolic outcomes, including reduced risk of post-transplant diabetes mellitus [41,42].

In our study, the FFQ-6 indicated the highest consumption frequency for vegetables and cereal products and the lowest for sweets/snacks, energy drinks, and alcoholic beverages. Dairy products and eggs, as well as meat and fish, were consumed relatively frequently. Previous studies have shown that low adherence to the Mediterranean diet and a high contribution of saturated fatty acids (SFA) are associated with higher BMI (>25 kg/m^2^) and increased graft-related risk, whereas dietary patterns characterized by higher protein intake and lower simple sugar consumption have been associated with reduced progression to overweight and improved allograft survival [9,43].

In another study, higher protein intake in the early post-transplant period (dietary protein intake ≥ 1.4 g/kg/day) was associated with better graft function at 3 months and a more favorable clinical profile [8].

From a clinical perspective, our findings suggest the importance of structured dietary monitoring beginning in the early post-transplant period. Unrestricted dietary intake after transplantation may be associated with excessive energy consumption, which is difficult to control and may contribute to progressive weight gain. This effect may be further amplified by immunosuppressive therapy, which is associated with increased appetite, metabolic alterations, and fat accumulation. Therefore, regular nutritional assessment and individualized dietary counseling should be considered essential components of post-transplant care to potentially mitigate adverse changes in body composition and reduce long-term cardiovascular risk.

However, increasing attention has recently been directed toward the nutritional status of patients prior to organ transplantation. Pre-transplant nutritional status may significantly influence post-transplant outcomes, indicating that early nutritional optimization could contribute to improved recovery and long-term clinical prognosis.

Potential strategies to optimize nutritional status during the waiting period for kidney transplantation in candidates on the transplant list include routine nutritional screening and comprehensive assessment. Management should involve individualized medical nutrition therapy to ensure adequate energy and protein intake, correction of micronutrient deficiencies (e.g., vitamin D, iron), and control of CKD-related metabolic abnormalities, including hyperphosphatemia and dyslipidemia. In addition, prehabilitation strategies combining nutritional support with structured physical activity may improve functional status and body composition, thereby potentially enhancing post-transplant clinical outcomes [44].

### 4.10. Limitations of the Study

Given the cross-sectional design of the study, all observed relationships should be interpreted as associations rather than causal effects. Therefore, several limitations of this study should be acknowledged. First, the cross-sectional design precludes conclusions regarding causality between dietary intake, anthropometric indices, and metabolic outcomes. Second, the relatively small sample size from a single transplant center limits the generalizability of the findings to broader kidney transplant populations.

Dietary intake was assessed using a 24 h dietary recall and the FFQ-6 questionnaire, both based on self-reported data and therefore subject to recall bias and reporting inaccuracies. In addition, a single 24 h recall may not fully reflect habitual dietary intake. Although multiple anthropometric indices were applied, direct body composition assessment methods (e.g., DXA or bioelectrical impedance analysis) were not used, which limits the precision of muscle mass and fat mass evaluation.

Furthermore, the heterogeneity of the study group with respect to time since transplantation and immunosuppressive regimens may have influenced the observed associations. Future longitudinal studies with larger, multicenter cohorts and objective body composition measurements are warranted to confirm these findings.

## 5. Conclusions

Kidney transplant recipients frequently exhibited unfavorable body composition and dietary imbalances that were not adequately captured by BMI alone. Despite mean BMI values within the upper normal range, a high prevalence of central adiposity was observed, particularly in older patients. Age was positively associated with multiple indices of visceral fat accumulation, emphasizing the limitations of BMI as a sole marker of nutritional status. Protein intake was positively correlated with calf circumference, which may indicate a potential association with skeletal muscle mass preservation. In addition, excessive sodium intake and insufficient intake of potassium, calcium, vitamin D, and unsaturated fatty acids were common. These findings suggest the need for comprehensive nutritional assessment and individualized dietary counseling to reduce metabolic risk and support long-term outcomes in kidney transplant recipients.

## Figures and Tables

**Table 1:** Data obtained in the study population.

Parameters	Mean ± SD	Range and Median
BMI (kg/m^2^)	24.9 ± 4.34	16.9–37.1 (24.7)
Age (years)	49 ± 14.3	19–77 (49)
Body weight (kg)	73.3 ± 13.43	41.2–114 (74)
WHR	0.96 ± 0.10	0.7–1.4 (0.9)
WHtR	54.4 ±7.28	36.7–77.5 (54)
WHt0.5R	7.1 ± 0.97	4.9–9.8 (7.0)
ABSI	0.083 ± 0.007	0.062–0.100 (0.083)
AVI	17.4 ± 4.77	7.9–30.8 (16.6)
BRI	4.25 ± 1.60	1.20–10.00 (4.08)
CI	1.30 ± 0.12	0.96–1.54 (1.29)
BAI	25.2 ± 6.32	9.2–47.2 (24.5)
RPI	41.2 ± 2.53	35.1–46.5 (41.2)
Calf circumference [cm]	35.53 ± 3.40	28–43 (35)
Waist circumference [cm]	92.46 ± 12.88	63–124 (91)
Arm circumference [cm]	28.19 ± 3.85	16–37 (28)
Hip circumference [cm]	96.46 ± 11.07	63–132 (98)
Time since transplantation (months)	28.8 ± 52.17	0.23–228 (2)
Duration of dialysis before transplantation (years)	2.9 ± 3.06	0–19 (2)
Estimated glomerular filtration rate (eGFR) CKD–EPI [mL/min/1.73 m^2^]	45.9 ± 18.87	9–89 (45)

**Table 2:** Anthropometric measures and protein intake in female and male groups.

Women	Mean ± SD Median (IQR)	Man	Mean ± SD Median (IQR)
BMI [kg/m^2^]	25.36 ± 5.36 24.7	BMI [kg/m^2^]	24.55 ± 3.37 24.7
Body weight [kg]	67.69 ± 14.33 67	Body weight [kg]	77.91 ± 11.02 79
Calf circumference [cm]	34.87 ± 4.04 35	Calf circumference [cm]	36.07 ± 2.76 36
Waist circumference [cm]	90.03 ± 15.69 90	Waist circumference [cm]	94.46 ± 10.01 94
Arm circumference [cm]	27.96 ± 4.57 29	Arm circumference [cm]	28.38 ± 3.26 28
Hip circumference [cm]	97.68 ± 12.61 98.5	Hip circumference [cm]	95.46 ± 9.84 98
ABSI	0.082 ± 0.008 -	ABSI	0.084 ± 0.007 -
AVI	16.6 ± 5.80 -	AVI	18.0 ± 3.78 -
BRI	4.54 ± 2.06 -	BRI	4.00 ± 1.09 -
CI	1.28 ± 0.14 -	CI	1.31 ± 0.10 -
BAI	28.8 ± 6.47 -	BAI	22.2 ± 4.47 -
RPI	40.4 ± 2.89 -	RPI	41.8 ± 2.05 -
WHt0.5R	7.0 ± 1.22 -	WHt0.5R	7.0 ± 0.73 -
Daily protein requirement [g]	57.03 ± 3.59 57.6	Daily protein requirement [g]	70.38 ± 5.80 71.1
Actual protein intake [g]	70.90 ± 24.25 65.3	Actual protein intake [g]	77.29 ± 23.94 77.9

**Table 3:** Independent Samples *t*-Test.

Variables	t	*df*	*p*
body shape index (ABSI)	−1.135	69	0.260
abdominal volume index (AVI)	−1.182	69	0.241 ^a^
body roundness index (BRI)	1.413	69	0.162 ^a^
conicity index (CI)	−0.941	69	0.350
body mass [kg]	−3.394	69	0.001
body adiposity index (BAI)	5.102	69	<0.001 ^a^
BMI [kg/m^2^]	0.772	69	0.443 ^a^
waist to height0.5 ratio (WHt0.5R)	−0.139	69	0.890 ^a^
reciprocal ponderal index (RPI)	−2.385	69	0.020 ^a^

^a^ Levene’s test is significant (*p* < 0.05), suggesting a violation of the equal variance assumption.

**Table 4:** Analysis of body mass index according to the type of diabetes.

Type of Diabetes	BMI Mean ± SD
Type 1 diabetes (*n* = 4)	21.15 ± 1.40
Type 2 diabetes (*n* = 3)	28.33 ± 3.23
PTDM (*n* = 18)	25.4 ± 4.99
Patients without diabetes (*n* = 45)	24.81 ± 4.23

PTDM—post-transplant diabetes mellitus.

**Table 5:** Frequency of consumption of different food groups.

FFQ 6—Food Groups	Mean ± SD (Range)
Sweets and snacks	2.3 ± 0.72 (1–6)
Dairy products and eggs	3.05 ± 0.78 (1–6)
Cereal products	2.71 ± 0.60 (1–6)
Fats	2.5 ± 0.70 (1–6)
Fruits	2.7 ± 0.77 (1–6)
Vegetables and grains	3.42 ± 0.68 (1–6)
Meat and fish products	2.92 ± 0.79 (1–6)
Beverages	2.4 ± 1.10 (1–6)
Energy drinks, vodka, alcoholic mixed drinks	1.2 ± 0.54 (1–6)

**Table 6:** Intake of energy and nutrients in the study group.

Nutrient	Mean ± SD	Range	Reference Intake *
Energy (kcal/day) kcal/kg/day	1596.75 ± 533.65 (21.87 ± 7.3)	837.9–3330.0	2013.71 ± 402.49 (30–35 kcal/kg/day)
Protein (g/day) g/kg/d	75.3 ± 23.05 (22.1% of energy) 1.03 ± 0.31	28.0–158.0	10–20% of energy 0.7–1.0 g/kg/day
Plant protein (g/day)	24.69 ± 12.60	6.4–74.5	~35% of total protein
Animal protein (g/day)	44.03 ± 17.4	2.5–92.0	~65% of total protein
Fat (g/day)	57.2 ± 25.0 (16.8% of energy)	20.6.5–145.3	25–35% of energy
Carbohydrates (g/day)	207.07 ± 82.36 (61.0% of energy)	96.0–472.0	45–65% of energy
Dietary fiber (g/day)	20.33 ± 10.66	3.8–75	20–25
SFA (g/day)	22.61 ± 10.51	6.2–59.8	≤22
MUFA (g/day)	18.95 ± 10.79	3.7–54.7	27–44
PUFA (g/day)	7.74 ± 4.43	0.9–24.2	13–24
Cholesterol (mg/day)	296.89 ± 243.45	22.5–1242	200–300
Sugar (g/day)	49.21 ± 32.15	0.3–211	≤50
Iron (mg/day)	10.42 ± 6.11	3.5–52	6–18
Potassium (mg/day)	2430.74 ± 917.634	514–4793.0	3500
Calcium (mg/day)	530.54 ± 344.55	61.5–2380.0	800–1200
Phosphorus (mg/day)	1045.39 ± 344.3	412.0–2361	580–700
Sodium (mg/day)	2025.52 ± 874.99	722.0–5060.0	<1500–2000
Magnesium (mg/day)	253.48 ± 121.31	62.0–754.0	255–420
Zinc (mg/day)	8.05 ± 3.0	2.6–24.6	6.8–11
Vitamin A (µg/day)	956.48 ± 706.81	25.1–3373.0	500–900
Vitamin C (mg/day)	102.47 ± 94.99	9.1–378	60–90
Vitamin D (µg/day)	1.63 ± 1.37	0.1–6.1	15
Vitamin E (mg/day)	8.30 ± 5.09	1.7–27.0	8–10
Vitamin K (µg/day)	9.05 ± 14.94	0.0–72.8	55–65
Vitamin B3 (mg/day)	17.40 ± 8.2	1.6–48.0	11–16
Vitamin B9 (µg/day)	294.85 ± 292.65	24.2–2461.0	320–400
Vitamin B12 (µg/day)	2.27 ± 1.28	0.1–5.7	2.0–2.4
Vitamin B1 (mg/day)	1.05 ± 0.44	0.1–2.2	0.9–1.3
Vitamin B2 (mg/day)	1.28 ± 0.51	0.1–2.7	0.9–1.3

* Dietary Reference Intakes for the Polish Population—2024.

**Table 7:** Intake of energy and selected nutrients according to type of diabetes.

Mean ± SD of Selected Nutrients According to Type of Diabetes								
Type of Diabetes	Energy Intake [kcal]	Protein [g]	Fat [g]	Carbohydrates [g]	Calcium [mg]	Sodium [g]	Potassium [mg]	Phosphorus [mg]
PTDM	1758.9 ± 544.7	78.8 ± 21.8	61.2 ± 29.1	225.6 ± 84.1	501.1 ± 269.5	2136.4 ± 934.8	2440 ± 1187.5	1009.4 ± 347.3
Type 1 diabetes	1399.8 ± 347.3	60.8 ± 9.3	46.4 ± 28.7	191.2 ± 19.5	337.5 ± 163.2	2315.5 ± 592.6	1981 ± 529.7	954.3 ± 239.1
Type 2 diabetes	1341.7 ± 292.9	70.8 ± 28.7	45.1 ± 28.9	171.3 ± 36.8	536.8 ± 262.4	1830.9 ± 707.0	2426.9 ± 171.03	1041.5 ± 137.4
Without diabetes	1543.4 ± 580.7	74.2 ± 25.9	54.4 ± 24.02	196.0 ± 90.9	547.0 ± 392.4	1944.7 ± 915.3	2685.7 ± 1565.4	1057.5 ± 368.1

## Data Availability

The data presented in this study are available on reasonable request from the corresponding author. The data are not publicly available due to privacy and ethical restrictions.
